# Comparative transcriptome analysis of melon (*Cucumis melo* L.) reveals candidate genes and pathways involved in powdery mildew resistance

**DOI:** 10.1038/s41598-022-08763-3

**Published:** 2022-03-23

**Authors:** Zengqiang Zhao, Yongmei Dong, Jianyu Wang, Guoli Zhang, Zhibin Zhang, Aiping Zhang, Zhijun Wang, Panpan Ma, Youzhong Li, Xiying Zhang, Chunxiu Ye, Zongming Xie

**Affiliations:** 1 Xinjiang Production & Construction Group Key Laboratory of Crop Germplasm Enhancement and Gene Resources Utilization, Biotechnology Research Institute, Xinjiang Academy of Agricultural and Reclamation Science, Shihezi, 832000 Xinjiang People’s Republic of China; 2 Agricultural Science Research Institute, The Sixth Division of Xinjiang Production & Construction Group, Wujiaqu, 831300 People’s Republic of China; 3 State Key Laboratory of Cotton Biology, Institute of Cotton Research, Chinese Academy of Agricultural Sciences, Anyang, 455000 People’s Republic of China; 4 Xinjiang Agricultural University, Urumqi, 830052 Xinjiang People’s Republic of China

**Keywords:** Computational biology and bioinformatics, Immunology, Plant sciences

## Abstract

Powdery mildew is a major disease in melon, primarily caused by *Podosphaera xanthii* (*Px*). Some melon varieties were resistant to powdery mildew, while others were susceptible. However, the candidate genes associated with resistance and the mechanism of resistance/susceptibility to powdery mildew in melon remain unclear. In this study, disease-resistant melon cultivar TG-1 and disease-susceptible melon cultivar TG-5 were selected for comparative transcriptome analysis. The results suggested that the numbers of differentially expressed genes (DEGs) in TG-5 was always more than that in TG-1 at each of the four time points after *Px* infection, indicating that their responses to *Px* infection may be different and that the active response of TG-5 to *Px* infection may be earlier than that of TG-1. Transcription factors (TFs) analysis among the DEGs revealed that the bHLH, ERF, and MYB families in TG-1 may play a vital role in the interaction between melon and powdery mildew pathogens. GO enrichment analysis of these DEGs in TG-5 showed that the SBP, HSF, and ERF gene families may play important roles in the early stage of melon development after *Px* infection. Finally, we speculated on the regulatory pathways of melon powdery mildew and found PTI and ABA signaling genes may be associated with the response to *Px* infection in melon.

## Introduction

Melon (*Cucumis melo* L.), a member of the Cucurbitaceae family, is an important economic crop grown worldwide^1^. However, melon is susceptible to powdery mildew disease (PM) at all the developmental stages thereby leading to significant losses in its production^2^. PM is a fungal disease in melon caused by *Golovinomyces cichoracearum* (Gc) or *Podosphaera xanthii* (*Px*) that can occur throughout the year. Infection by the pathogen disrupts the photosynthetic activity of the leaves, resulting in impaired plant growth and reduced yields and fruit quality^3^. *Px* is the predominant causal agent of PM in melon, which could result in the decline of the yield and quality of melon^2,4^. In the last few decades, many studies were conducted to identify candidate/functional genes for powdery mildew resistance by using forward genetics approaches^3,5–10^, while some other studies were employed by reverse genetics approaches^11–13^.

RNA sequencing (RNA-Seq), as a revolutionary technique for transcriptome analysis, has been widely used to identify core pathways and responsive genes under biotic or abiotic stress. It provides a precise measurement for transcript levels to reveal the response mechanisms for specific stimuli. For instance, disease stress-inducible pathways and their corresponding genes have been investigated in *Arabidopsis*^14^, *Capsicum annuum*^15^, and wheat^16^ by using comparative transcriptome analysis. Recently, transcriptomic studies on melon resistance to PM have gradually become a hot topic for melon. For example, Cheng et al. selected one melon variety with thin rinds ‘GanTianmi’ and another varietal with thick rinds ‘XueLianHua’ to conduct comparative transcriptome analysis and further identify some candidate DEGs and TFs related to disease resistance, such as MYB, ERF and WRKY^17–20^. Moreover, Gao et al. compared the lncRNAs between susceptible and resistant melon cultivars in response to PM infection^21^. Zhu et al. also analyzed the comparative transcriptome of the melon resistant MR-1 and susceptible Topmark cultivars to identify candidate genes^17–19,21,22^. Extensive studies have reported that loss-of-function mutations in one or more appropriate mildew resistance locus (MLO) genes can protect plants from powdery mildew fungi infection^23^. Lovieno et al. have identified 16 MLO homologs genes in *C. melo*^24^. Howlader et al*.* reported the expression of 14 *CmMLO* genes after infection of Melon with seven different races of *P. xanthii*^25^.

In this study, a comparative transcriptome analysis of the resistant cultivar TG-1 and the susceptible cultivar TG-5 was performed to identify some candidate DEGs related to powdery mildew resistance. These results provided several new insights into the molecular defense mechanisms of melon cultivars exhibiting strong resistance to *Px* infection and valuable information for breeding powdery mildew resistant melon cultivars.

## Materials and methods

### Plant maintenance and pathogen infection

**Figure 1:**
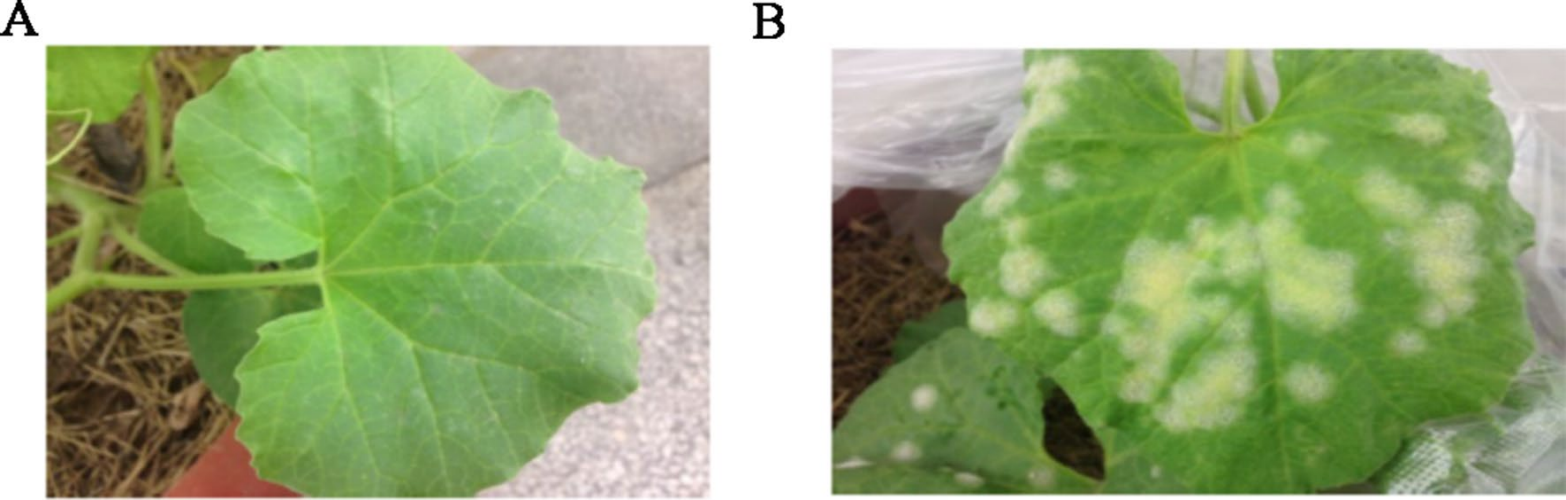
Different phenotypes of resistant and susceptible melon cultivars were observed after 5 days of powdery mildew infection. (**A**) TG-1 (powdery mildew resistant); (**B**) TG-5 (powdery mildew susceptible).

*Cucumis Melo* cultivars TG-1 (powdery mildew resistant) and TG-5 (powdery mildew susceptible) were used for inoculation with powdery mildew in this study (Fig. 1). TG-1 and TG-5 were grown in a greenhouse under a controlled temperature of ~ 28 ℃/~ 22 ℃ (day/night), a 14 h/10 h light/dark, and an average of 60% humidity. At the 3–5 leaves stage and when plants were approximately 15–20 cm high, three plants of each cultivar were inoculated with powdery mildew by cutting the leaf blades at mid-length with sterile scissors previously dipped in the spore suspension. Three plants of each cultivar were mock-inoculated with sterile liquid medium to use as negative controls. To clarify the changes in the gene expression levels of TG-1 and TG-5 during the first stages of their compatible interactions with *Px*, leaf samples distal from the wound site were taken before infection (0 days, control) and after 1, 2, 3 and 5 days, respectively. Three biological replicates were prepared at each time point. In total, 30 samples were immediately frozen in liquid nitrogen and stored at − 80 ℃ for total RNA isolation and further analysis.

### RNA extraction, library construction and RNA sequencing

Total RNA was extracted using TRIZOL reagent (Invitrogen, Gaithersburg, MD, USA) and purified with an RNeasy mini kit (QIAGEN, Germantown, MD, USA) as described by the manufacturer's instruction. the Qubit RNA Assay Kit in a Qubit 2.0 Fluorometer (Life Technologies, CA, USA) was used to measure the RNA concentration. High-quality RNA was then used for library construction using the Illumina TruSeq Stranded RNA Kit (Illumina, San Diego, CA, USA) following the manufacturer's instructions. The purified cDNA libraries were further enriched by PCR. Transcriptome sequencing of the prepared libraries was performed on an Illumina 2500 platform with paired-end 150 bp reads (Novogene Bioinformatics Institute, Beijing, China).

Total 30 cDNA libraries were generated using the Illumina HiSeq 2500 platform in this study. All raw reads were assessed for quality using the program of FastQC (V0.11.3) (https://www.bioinformatics.babraham.ac.uk/projects/fastqc/) with the default parameters and filtered using Trimmomatic (V0.38)^26^ (parameters: ILLUMINACLIP: TruSeq3-PE-2.fa:2:30:10 SLIDINGWINDOW:5:20 LEADING:5 TRAILING:5 MINLEN:50) to acquire clean data. Then clean reads of all samples were aligned to the reference genome V3.6.1 of melon (*Cucumis melo* L.) (ftp://cucurbitgenomics.org/pub/cucurbit/genome/melon/v3.6.1/CM3.6.1_pseudomol.fa.gz)^1^ by using Hisat2 (v2.0.5)^27^ with the default parameters. The HTSeq^28^ software with the default parameters was used to count the number of RNAseq reads that mapped to each gene of *C. melo* reference. The count files were then merged into a count table containing read-count information for all samples. DESeq2 V1.30.1^29^ was subsequently applied to the count table separately to calculate the gene expression levels. We measured the expression levels with log2-transformed expression values. To allow log2-transformation of genes with expression values of zero, we added 0.01 to the expression values before log2-transformation.

### Identification of differentially expressed genes and GO enrichment analysis

Differential gene expression analysis between pairs of samples was performed on the normalized data obtained above using the DESeq2 V1.30.1^29^ package, and the adjusted *p*-values were calculated using the Benjamini and Hochberg method to control the false discovery rate. The R function prcomp was used to perform Principal Component Analysis (PCA) analysis on the expression matrix of all samples. The R package ggord V1.1.6^30^ was used to visualize the results of PCA. The standard for screening DEGs were set as *p*-values < 0.05 and Log_2_ (fold change) > 1 or <  − 1. We used two different grouping comparison methods to identify DEGs. The first grouping is the comparison between disease-resistant materials and susceptible materials on the first 1, 2, 3, and 5 days after the inoculation of the pathogens, and the susceptible materials are used as the control. The second grouping method is disease-resistant and susceptible cultivars at days 1, 2, 3 and 5 were compared with those expressed in the respective control group (day 0, sterile liquid medium) to find DEGs. Trend analysis is a method of clustering gene expression patterns (the shape of the expression profile over multiple phases) for multiple "continuous" samples using read counts by STEM^31^ software with the default parameters. Trend analysis was used to divide the different time point expression patterns into differential clusters and thus find genes with the same expression pattern. It was applied to at least three and more consecutive type samples (samples containing a specific temporal, spatial or treatment dose size order, etc.). A trend with a *p*-value < 0.05 was considered as a significant trend and expressed as over-represented cluster. The GO annotations from the melon database (ftp://cucurbitgenomics.org/pub/cucurbit/genome/melon/v3.6.1/CM3.6.1_GO_anno.txt.gz) were downloaded and used the clusterProfiler^32^ package V3.18.0 for GO enrichment analysis. Heatmaps were drawn by R package heatmap V1.0.12.

### Transcription factor analysis

Plant transcription factors (TFs) were predicted on PlantTFDB v5.0^33^ (http://planttfdb.gao-lab.org/prediction.php) with *Arabidopsis thaliana* as a reference species. Each group of DEG-associated protein sequences was used to predict the TFs. The distribution of differentially expressed transcription factors was visualized by ggplot2 V3.3.3.

### Identify the MLO gene of *C. melo*

The *C. melo* MLO gene was obtained from the Melonomics melon genomic database (http://cucurbitgenomics.org/) according to the method of Ioveno et al*.*^24^, and found 18 *C. melo* MLO genes in total.

### Statement of compliance

Experimental material involves the research between Cucumis Melo cultivars TG-1 (powdery mildew resistant) and TG-5 (powdery mildew susceptible) complies with institutional, national, and international guidelines and legislation.

## Results

### Transcriptome analysis

To obtain a comprehensive understanding of gene expression dynamics in leaf tissues of *Cucumis Melo* after *Px* infection, cDNA libraries from a total of 30 samples of resistant and susceptible cultivars were subjected to transcriptome sequencing at 0, 1, 2, 3, and 5 days post *Px* infection. The resistant cultivar TG-1 is denoted by RC, RT1, RT2, RT3 and RT5, respectively, where R stands for resistance, C stands for control and the number indicates the sampling time post Px infection. The susceptible cultivar TG-5 is denoted by SC, ST1, ST2, ST3 and ST5, respectively, where S represents susceptible, C represents control, and the number indicates the sampling time post *Px* infection (Table S1). Correlation analysis showed that the three biological replicates from each treatment were correlated (Fig. S1A). The PCA analysis of the expression matrix of all samples showed that PC1 can explain 33.66% of the grouping variance (Fig. S1B).

### Identification and annotation of differentially expressed genes

**Figure 2:**
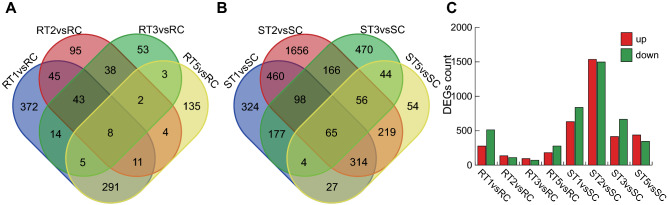
Differentially expressed genes (DEGs) across four time points after *Px* infection in resistant (TG-1) and susceptible cultivars (TG-5). (**A**) Venn diagram of DEGs in the resistant melon cultivar at each of four time points after *Px.* infections. (**B**) Venn diagram of DEGs in the susceptible melon cultivar at each of four time points after *Px* infections. (**C**) Distribution of up-regulated and down-regulated gene expression patterns across the four time points after *Px* infections in resistant and susceptible cultivars.

Compared to control plants inoculated with sterile liquid medium (SC and RC), DEGs were identified in both cultivars at different time points post *Px* inoculation (Fig. 2). Total 6366 DEGs were found in susceptible cultivar TG-5 and total of 1660 DEGs in resistant cultivar TG-1 in four treatment groups (Fig. 2, Table S2). 277, 136, 95 and 181 DEGs were up-regulated at 1, 2, 3, and 5 days in TG-1, respectively. 631, 1536, 415 and 438 DEGs were up-regulated at 1, 2, 3, and 5 days in TG-5, respectively (Fig. 2A–C). A total of 512, 110, 71 and 278 DEGs were down-regulated at 1, 2, 3, and 5 days in TG-1, respectively. 838, 1498, 665, and 345 DEGs were down-regulated at 1, 2, 3, and 5 days in TG-5, respectively. During the 1–5 days sampling period, the number of DEGs in TG-5 was higher than that in TG-1. Moreover, genes down-regulated were more than that up-regulated in both TG-1 and TG-5 (Fig. 2C, Fig. S2). And 8 DEGs were identified at all four time points in TG-1 (Fig. 2A). The main functions of these 8 genes are Peroxidase, Xyloglucan endotransglucosylase, Aspartate-tRNA ligase, Short-chain dehydrogenase, 1-deoxy-d-xylulose-5-phosphate synthase and transcription factor BEE 3-like (Table S3).

In addition, DEGs were also analyzed between the two cultivars at the same time points after Px inoculation. Amounts of 1294, 783, 4941 and 1317 DEGs were specifically expressed in the RT1 vs ST1, RT2 vs ST2, RT3 vs ST3 and RT5 vs ST5 comparisons, respectively (Table S4). Finally, a total of 22 DEGs were expressed in all four comparisons (Fig. S3). We identified 18 *C. melo* MLO genes, including the 15 MLO genes reported by Ioveno et al*.*^24^. Of all the DEGs obtained above, a total of four differentially expressed MLO genes were detected in RT3 vs ST3 and RT5 vs ST5 (Table S5). During the first two days, no differentially expressed MLO genes were identified. The MLO genes *MELO3C019435* and *MELO3C016709* were up-regulated in the resistant cultivar TG-1 compared to the susceptible cultivar TG-5 at 3 days post *Px* infection (RT3 vs ST3), while *MELO3C025761* and *MELO3C025760* were down-regulated at 5 days post *Px* infection (RT5 vs ST5).

### Gene expression patterns analysis and DEGs functional classification

**Figure 3:**
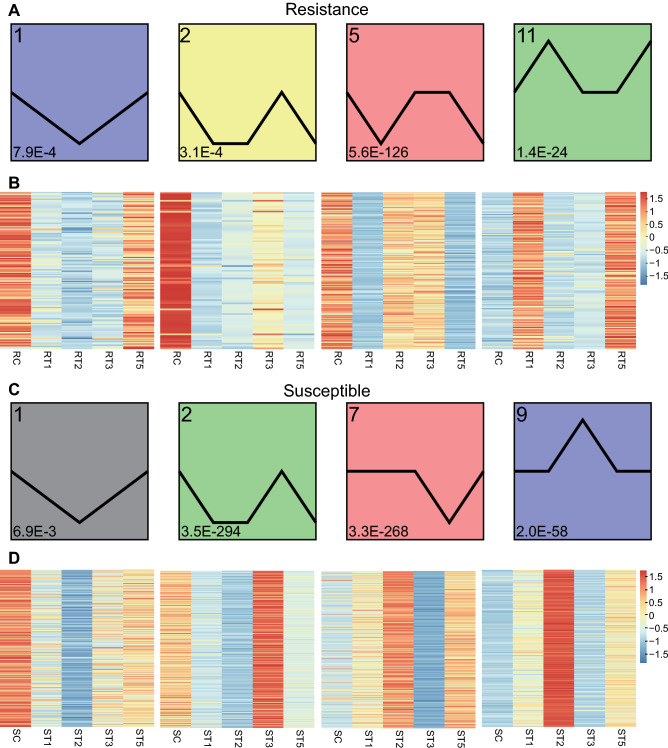
Trend analysis of gene expression in disease-resistant and disease-susceptible cultivars at different time points post infection. (**A**) 4 significant clusters of the resistant cultivar (*p*-value < 0.05). (**B**) Heatmap of the 4 significant clusters of the resistant cultivar. (**C**) 4 significant clusters of the susceptible cultivar (*p*-value < 0.05). (**D**) Heatmap of the 4 significant clusters of the susceptible cultivar. The heatmap colouring reflects the rank of the Z-scores. Data normalized by Z-score transformation can be used directly in the calculation of significant changes in gene expression between different samples and conditions.

Gene expression trend analysis showed that all DEGs identified in TG-1 and TG-5 across the different time points were clustered into 16 profiles, respectively (Fig. S2). In TG-1, 735 (67.7%) DEGs showed apparent changes for expression levels post *Px* infection and were grouped into four over-represented clusters (1, 2, 5 and 11; *p* < 0.05), and 532 DEGs were clustered into profiles 1, 2, and 5 and were mostly down-regulated post *Px* infection (Fig. 3A,B; Fig. S4A). In TG-5, 75.9% (3067) of the DEGs were grouped into four over-represented clusters (1, 2, 7 and 9; *p* < 0.05), and profiles 1, 2, and 7 were mostly down-regulated after *Px* infection, except cluster 9 which exhibited a trend of up-regulation (Fig. 3C,D; Fig. S4B, Table S6). In addition, the Venn results showed that some genes in cluster1, 2, 5 and 11 of TG-1 were also present in cluster1, 2, 7 and 9 of TG-5, respectively, indicating that these genes have different expression trends in two cultivars (Fig. S5, Table S7).

**Figure 4:**
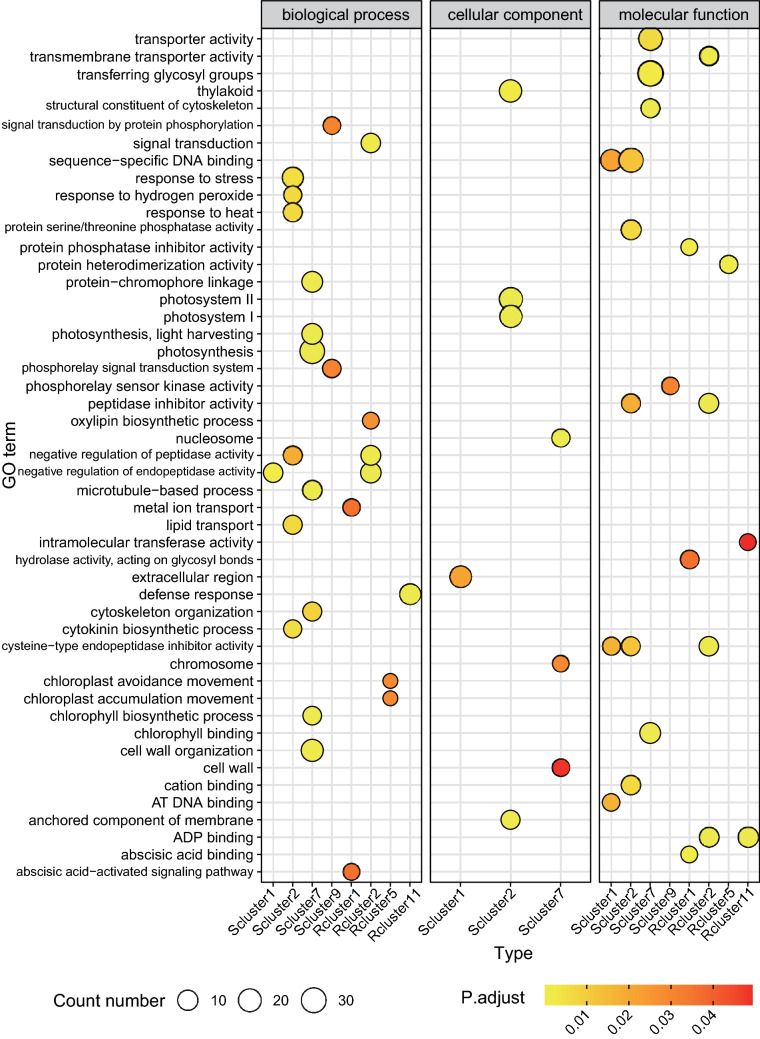
GO enrichment analysis of 8 significant clusters of DEGs associated with disease resistance and susceptibility (only the top 15 GO terms in each cluster are shown). The abscissas Scluster1 and Rcluster2 represent cluster 1 of susceptible materials (TG-5) and cluster 2 of resistant materials (TG-1) in trend analysis, respectively. Count number means the genes count of the GO term. The P. adjust means the adjust P-value of GO enrichment analysis.

To further identify the function of the notable transcripts differentially expressed between the two cultivars under *Px* infection, we performed GO enrichment analysis of DEGs from the over-represented profile. In TG-5, the most abundant GO terms were negative regulation of endopeptidase activity (GO:0010951), cysteine-type endopeptidase inhibitor activity (GO:0004869), response to stress (GO:0006950), protein serine/threonine phosphatase activity (GO:0004722), and peptidase inhibitor activity (GO:0030414) in profiles 1 or 2, in which gene expression decreased from day 0 to 1, and low expression levels were maintained thereafter. Molecular functions analysis showed that these target genes were mainly enriched in enzyme activity (Fig. 4, Table S8). In TG-1, genes involved in protein phosphatase inhibitor activity (GO:0004864), hydrolase activity, acting on glycosyl bonds (GO:0016798), defense response (GO:0006952), intramolecular transferase activity (GO:0016866), negative regulation of peptidase activity (GO:0010466), peptidase inhibitor activity (GO:0030414), negative regulation of endopeptidase activity (GO:0010951), cysteine-type endopeptidase inhibitor activity (GO:0004869) and transmembrane transporter activity (GO:0022857) were enriched in clusters 1, 5, or 11. Interestingly, genes enriched in cluster 7 of TG-5 and cluster 2 of TG-1 mainly related to photosynthesis, such as photosystem I (GO:0009522), photosystem II (GO:0009523), photosynthesis (GO:0015979), photosynthesis and light-harvesting (GO:0009765), chlorophyll-binding (GO:0016168), chloroplast avoidance movement (GO:0009903) and chloroplast accumulation movement (GO:0009904) (Fig. 4, Table S8). Besides, the GO enrichment results for DEGs between TG-5 and TG-1 at each time point also remained mostly consistent with the above results (Fig. S6, Table S9), indicating that these DEGs were actively expressed after *Px* infection.

### Characterization of transcription factors among DEGs

**Figure 5:**
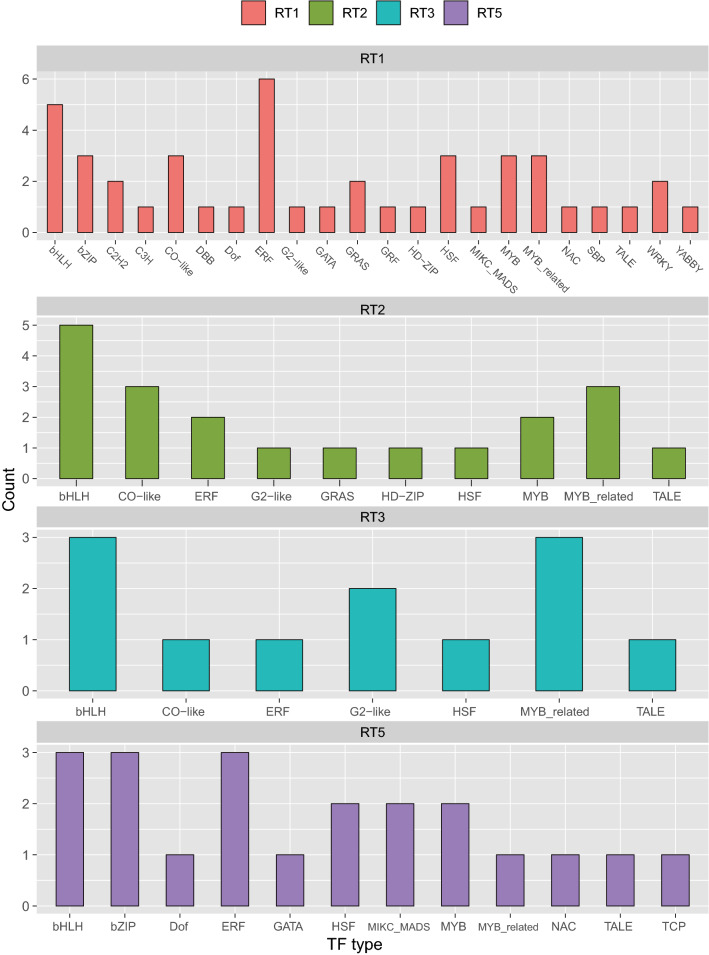
The distribution of transcription factor (TF) associated with disease resistance (TG-1). RT1 represents the transcription factor differentially expressed in the disease-resistant material (TG-1) on the first day after inoculation compared with that before inoculation.

A total of 536 TFs among the DEGs were identified in TG-1 and TG-5. Among them, 97 TFs were identified for TG-1, and a total of 439 TFs were identified for TG-5 (Table S10). The TF families that differentially expressed across the four time points in TG-1 were bHLH, ERF, MYB_related and TALE (Fig. 5). Enrichment analysis of differentially expressed transcription factors on day 1 (ST1) in TG-5 found these TFs were significantly enriched in the families SBP, HSF, and ERF (Fig. S7A). There were two differentially expressed transcription factors in common between the resistant material (TG-1) and the susceptible material (TG-5) in the four periods (Fig. S7B). The two genes, MELO3C004556 and MELO3C006431, belong to the HSF and ERF transcription factor families, respectively.

### Signal transduction pathways in response to Px infection

**Figure 6:**
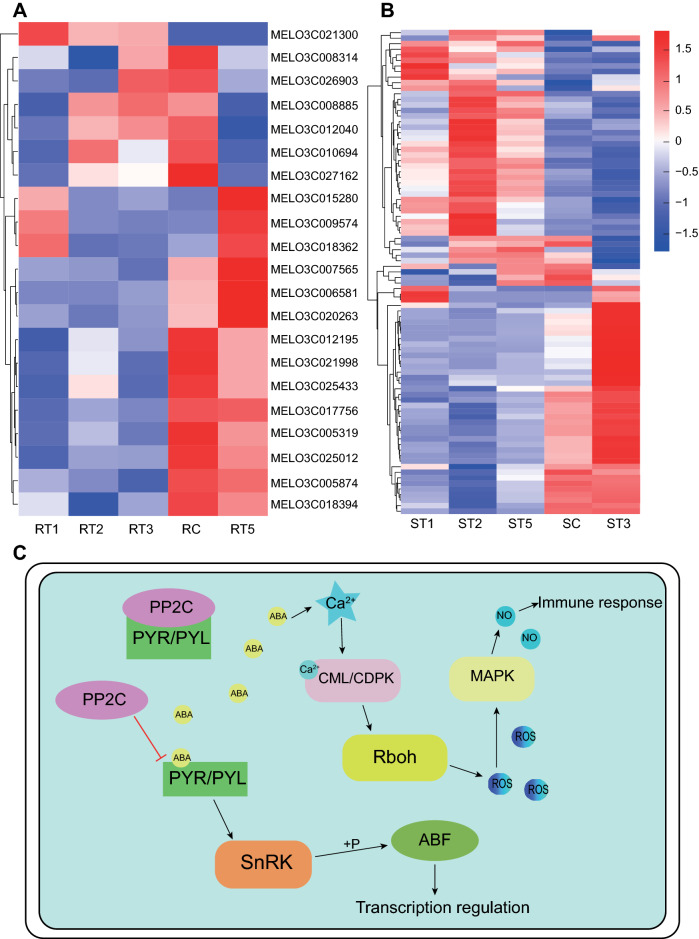
Regulatory pathways of disease resistance and susceptibility genes. Heatmap of expression levels of candidate disease resistance (**A**) and susceptible (**B**) genes post *Px* inoculation*.* (**C**) Pathway of disease resistance and susceptibility.

Phytohormones responsible for signal transduction can modulate systemic defense responses, such as PTI, ETIJA, SA, and ABA^18^, and play important roles in disease resistance. Based on these results, PTI and ABA signaling genes were found to be potentially involved in the reactions of TG-1 and TG-5 melon cultivars against the *Px* pathogen in this research. In the ABA signaling pathway, three genes encoding PYR/PYL proteins were down-regulated in TG-1 after *Px* infection. Genes encoding PYR/PYL and SnRK2 were down-regulated in TG-1 at all four time points, especially at 1 day post *Px* infection (Fig. 6A,C). In contrast, genes encoding SnRK2 in TG-5 were up-regulated at early stage after *Px* infection, then down-regulated at subsequent stages (Fig. 6B,C). In the PTI signaling pathway, seven genes encoding CML/CDPK, and one gene encoding MAPK were up-regulated in TG-1 at all four time points (Fig. 6A,C). In TG-5, eight CML/CDPK encoding genes, three Rboh encoding genes, and all four MAPK encoding genes were down-regulated after *Px* infection, while only four CML/CDPK encoding genes were up-regulated (Fig. 6B,C, Table S11).

## Discussion

In this study, comparative transcriptome and trend analysis revealed fundamental changes in gene expression patterns between resistant and susceptible melon cultivars at four different time points after inoculation with the fungal pathogen *Px*. Some GO terms, such as response to stress and defense response, exhibited a pattern in which the gene expression level was decreased at day 1 in TG-5 (cluster 2, Fig. 4), but increased at day 1 in TG-1 (cluster 1, Fig. 4), suggesting these gene functions may play vital roles in the resistance response to melon *Px* infection.

Although the MLO gene is an important weapon in the fight against powdery mildew^23^, none of the differentially expressed MLO genes of TG-5 and TG-1 both were differentially expressed in all four stages, and no SNPs were found in the MLO genes between the two cultivars. As Howlader et al*.*^25^ reported, MLO genes have different expression patterns after being infected by powdery mildew bacteria, and some are up-regulated and down-regulated. It is difficult to say that TG-1 resistance or TG-5 susceptibility is related to these differentially expressed MLO genes.

Systemic acquired resistance (SAR) is one of several induced defense responses in plants. It is regulated by plant hormones responsible for signal transduction and plays a vital role in disease resistance^34^. The phytohormone abscisic acid (ABA) plays a vital role in plant responses to biotic and abiotic stresses^35,36^. ABA receptors have three families of proteins, anti-Pyravbactin (PYR), anti-Pyravbactin-like (PYL) and ABA receptor regulatory components (RCAR), which form a complex to mediate ABA signaling^37,38^. Plant protein phosphatase 2C (PP2C) family members and SNF1-related protein kinase 2 (SnRK2) are key components of the ABA signal transduction pathway^39,40^. PP2C is a negative regulatory element that normally binds to the ABA receptor protein, leaving the ABA receptor protein in an inhibited state^41^. Once the plant is affected by adverse external factors and the intracellular ABA hormone level is elevated, ABA will bind to PYR/PYL and PP2C will release the ABA receptor protein, which in turn releases inhibition of PYR/PYL. PYR/PYL then mediates SnRK phosphorylation to activate the downstream transcription factor ABF to regulate cellular transcription. The phosphatase PP2C acts as a constitutive negative regulator of kinases (SnRK2) family when ABA is absent, whose autophosphorylation is required for the kinase activity of downstream targets^42–44^. We found that DEGs encoding the ABA receptor protein, such as gene *MELO3C018394*, showed an increasing expression trend after *Px* inoculation in the susceptible cultivar, while the opposite was true for the resistant cultivar. Expression of this gene was significantly lower at each time point in the susceptible cultivar than in the disease-resistant cultivar. More importantly, expression levels of the ABF-encoded genes (*MELO3C018458* and *MELO3C010850*) were significantly decreased on days 1 and 2 after *Px* inoculation in the susceptible cultivar, indicating that the transcript levels of susceptible material were significantly repressed by *Px* infection. These results indicate that the up-regulation of genes encoding ABA receptor was associated with the susceptibility of melon to this PM pathogen.

ABA also induces an increase in intracellular calcium ion concentration^20^. Ca^2+^ can be derived from intracellular calcium pools or extracellular sources, and Ca^2+^ usually acts as an intracellular secondary messenger that activates protective enzymes and improves photosynthesis to alleviate the damage caused by low or high temperatures, drought, high salinity and pests^45,46^. Currently, Ca^2+^ receptors in plants are divided into three major families: calcium-dependent protein kinase (CDPK), CaM-like protein (CML) and calcineurin B-like protein (CBL)^47,48^. When the intracellular Ca^2+^ concentration increases, it promotes Ca^2+^ binding by calcium-binding proteins thereby activating them, after which calcium-binding proteins indirectly activate NADPH oxidase (NOX) to generate reactive oxygen species (ROS), which further activate the MAPK cascade reaction^49,50^. Bivi et al*.* showed that sprayed calcium nitrate treatments significantly controlled the occurrence of stem rot in oil palms^51^. Madani et al*.* demonstrated that pre-harvest spraying of calcium chloride on papaya reduced the germination of anthracnose spores thus controlling disease incidence^52^. In this study, 9 DEGs within the disease-resistant cultivars encoded CML/CDPK proteins, of which 7 showed a decreasing expression trend at days 1 and 2, followed by a certain degree of increased expression for most of them. In the disease-susceptible cultivar, 16 DEGs encoded CML/CDPK proteins, 11 of which showed a decreasing expression trend and 5 showed an increasing trend at day 1, followed by most of them showing some degree of decrease after *Px* injection. On day 5 after *Px* injection, the expression levels of the DEGs *MELO3C012195* (7.11 in TG-1; 3.96 in TG-5) and *MELO3C015280* (11.79 in TG-1; 8.67 in TG-5), encoding CML/CDPK proteins, were higher in the disease-resistant cultivar than that in the disease-susceptible cultivar. Exhibiting a similar trend, expression of one DEG (*MELO3C007565*), encoding a MAPK protein, in the disease-resistant cultivar continued to decrease from the 1st to the 3rd day, after which it significantly increased on the 5th day after *Px* inoculation; 4 DEGs (*MELO3C007543*, *MELO3C009916*, *MELO3C020535*, *MELO3C006511*) encoding MAPKs were identified in the disease-susceptible cultivar showed a decreasing expression trend after which transcripts were sustained at a low expressions level. These results suggested that these up-regulated genes encoding CML/CDPK and MAPK proteins may contribute to the resistance response of melon to PM and the regulatory network of TG-1 in response to *Px* infection was more complex and diverse than that of TG-5. Utilizing effective defense pathways comprising a complex resistance network is necessary for melon in response to *Px* infection. Moreover, further investigations will be focused on functional validation of the selected DEGs, which could provide a helpful tool for the development of melon varieties resistant to *Px*.

## Conclusion

In this study, a total of 6366 and 1660 DEGs were identified in susceptible melon cultivar TG-5 and resistant melon cultivar TG-1 in four treatment groups after *Px* infection, respectively. Further analysis showed that 8 DEGs identified at all four time points in TG-1 were primarily involved in the xyloglucan metabolic process, hydrolase activity, and response to oxidative stress, which related to melon resistance to powdery mildew. Furthermore, GO enrichment analysis suggested that bHLH, ERF, and MYB TF families in TG-1, SBP, HSF, and ERF gene families in TG-5 may play a vital role in PM resistance.

## Supplementary Information

Supplementary Information.

## Data Availability

Raw data from this study were deposited in the NCBI SRA (Sequence Read Archive) database numbers PRJNA791790.
