# Supplementary material for: Comparative transcriptome analysis of melon (Cucumis melo L.) reveals candidate genes and pathways involved in powdery mildew resistance
Source: Sci Rep. 2022 Mar 23;12:4936. doi: 10.1038/s41598-022-08763-3 (PMC8943038; doi:10.1038/s41598-022-08763-3)
Supplement: Supplementary file 1 — Supplementary Information. [file 41598_2022_8763_MOESM1_ESM.zip › Supply Figure and Table/Supplyment Figure.docx]

**Supplementary Figure**


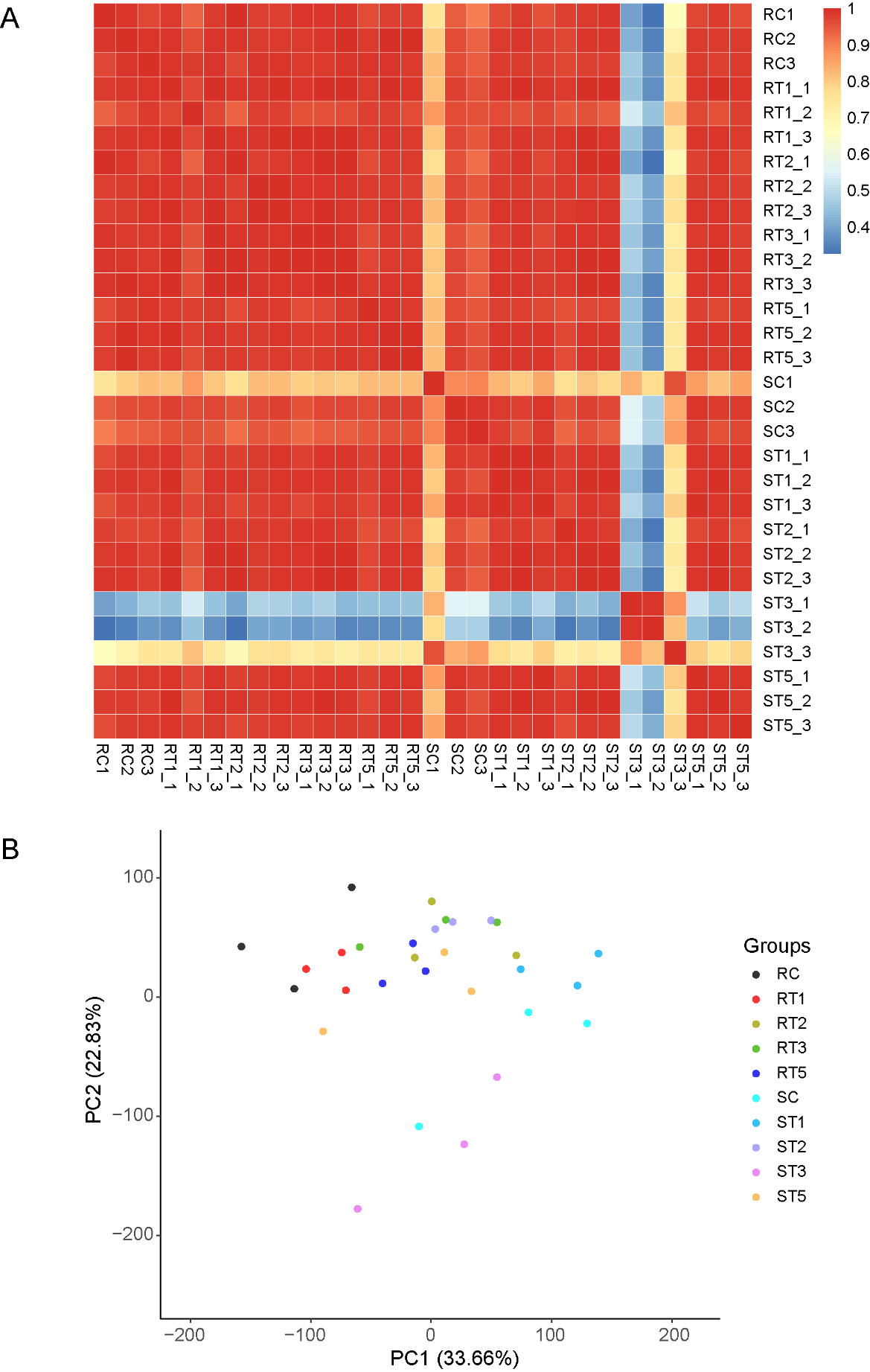


**Figure S1.** Correlation between samples of disease-resistant and susceptible cultivars.

(A) Correlation analysis of expression matrices for all samples. (B) PCA analysis of expression levels of all samples. Three biological replicates of each material are represented by the same color.


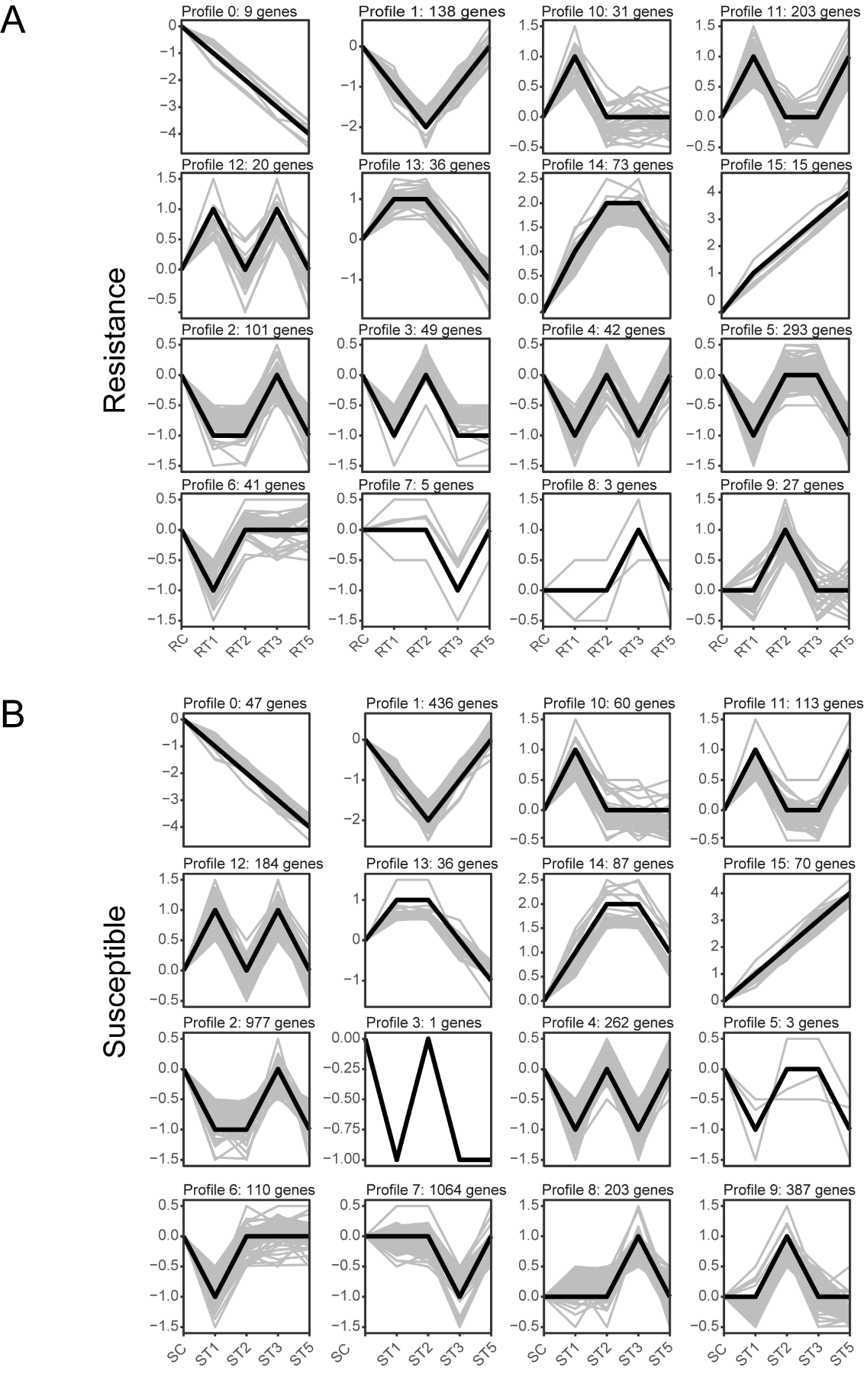


**Figure S2.** Detailed trend chart of disease-resistant and susceptible cultivars.

(A) Distribution of 16 clustered gene numbers of the disease-resistant cultivar. (B) Distribution of 16 clustered gene numbers of the disease-susceptible cultivar.

**
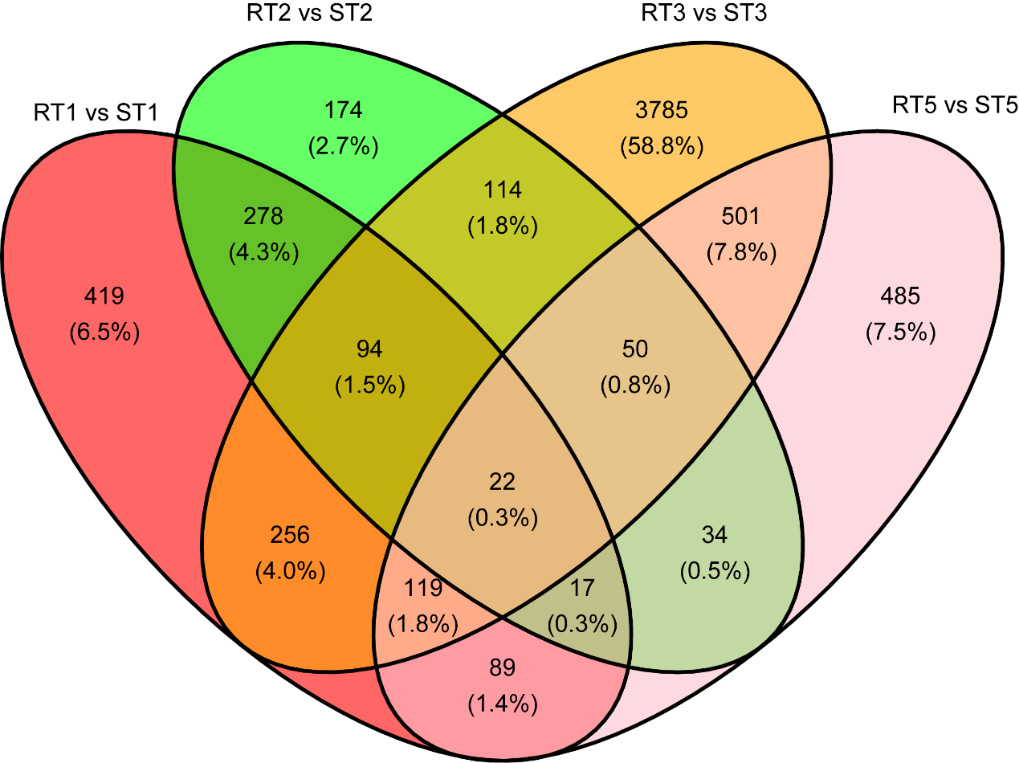
**

**Figure S3.** The venn diagram of the differential genes of disease-resistant and susceptible materials at 4 groups.

The abscissa RT1 vs ST1 represents the DEGs between resistant material (TG1) and susceptible material (TG5) on the first day after inoculation with bacteria, and so on. The numbers in the Venn diagram represent the number of DEGs, and the numbers in parentheses represent the percentage of the number of DEGs in the total number.

**
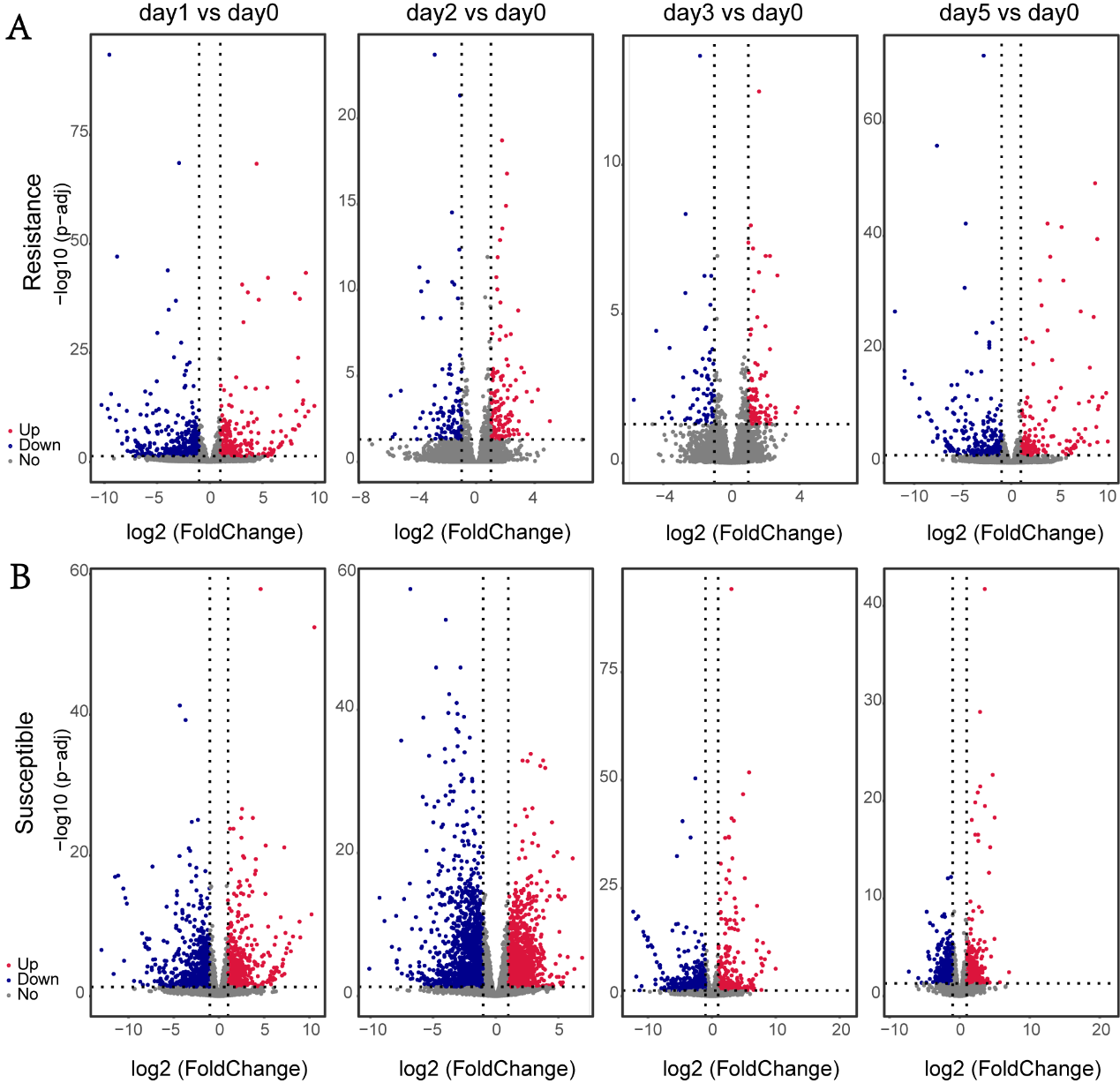
**

**Figure S4.** Volcano map of disease -resistant (A) and -susceptible (B) cultivars gene expression patters across 4 time points after *Px* inoculation.


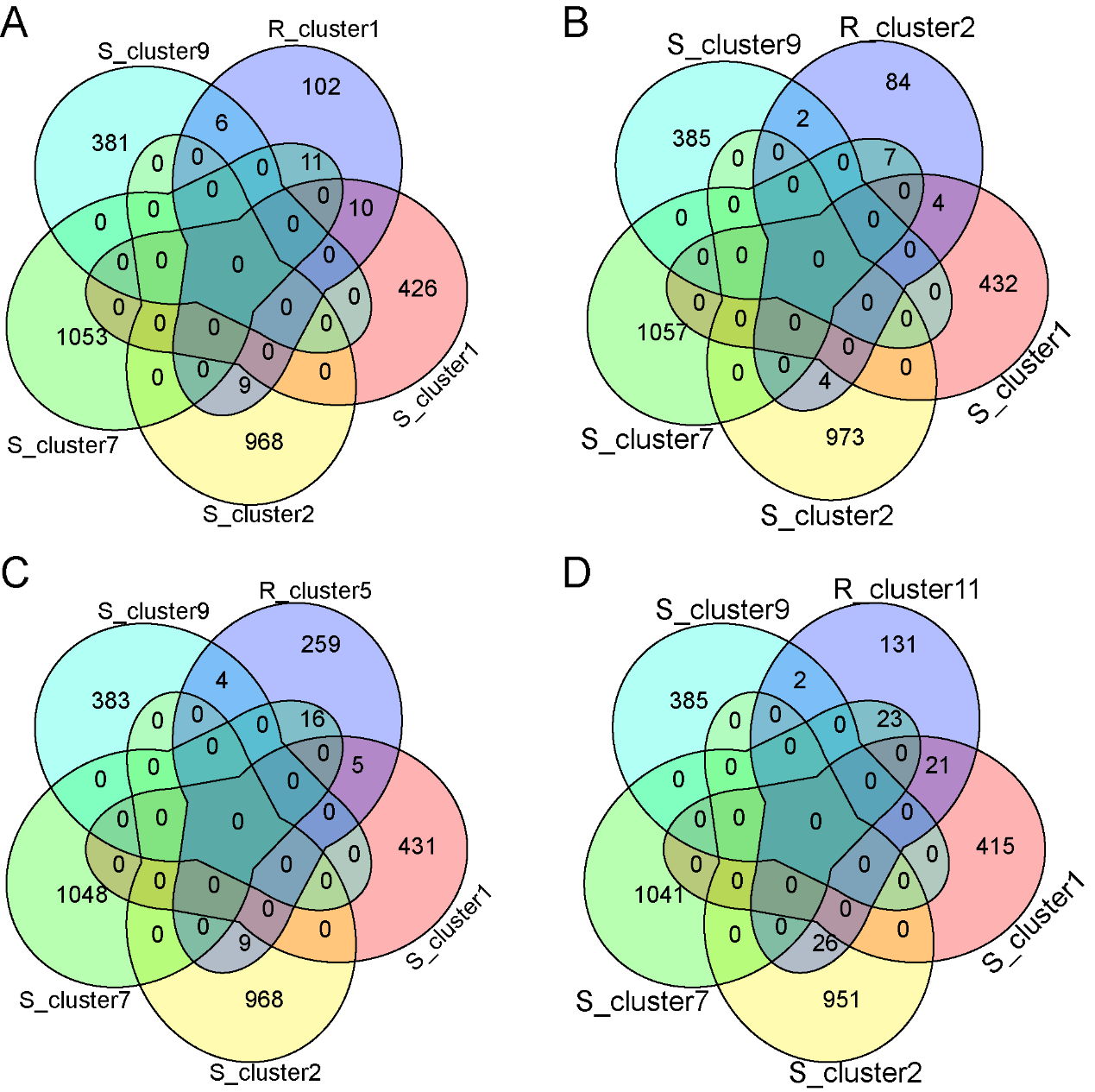


**Figure S5.** Comparison of differentially expressed genes (DEGs) in clusters 1, 2, 5 and 11 of resistant material (TG-1) with clusters 1, 2, 7 and 9 of susceptible material (TG-5).

(A) Venn diagram of DEGs in clusters 1 of TG-1 compared to DEGs in clusters 1, 2, 7 and 9 of TG-5. (B) Venn diagram of DEGs in clusters 2 of TG-1 compared to DEGs in clusters 1, 2, 7 and 9 of TG-5. (C) Venn diagram of DEGs in clusters 5 of TG-1 compared to DEGs in clusters 1, 2, 7 and 9 of TG-5. (D) Venn diagram of DEGs in clusters 11 of TG-1 compared to DEGs in clusters 1, 2, 7 and 9 of TG-5.


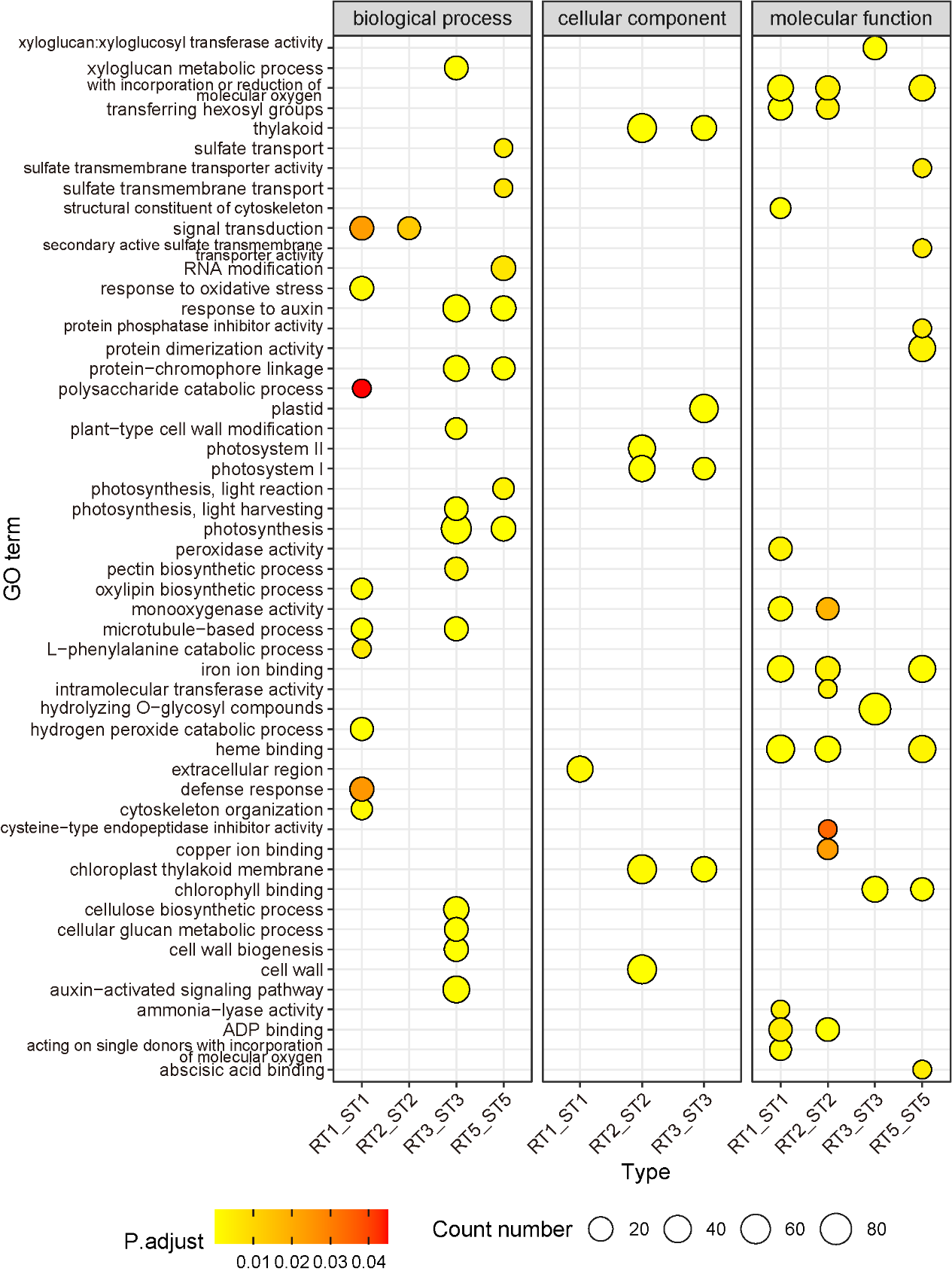


**Figure S6**. GO enrichment analysis of DEGs between the 4 groups of disease-resistant materials (TG1) and susceptible materials (TG5) (only top 20 GO terms in each cluster are displayed).

The abscissa RT1_ST1 represents the GO enrichment analysis of DEGs between TG1 and TG5 on the first day after inoculation with bacteria, and so on. Count number means the genes count of the GO term. The P. adjust means the adjust P-value of GO enrichment analysis.


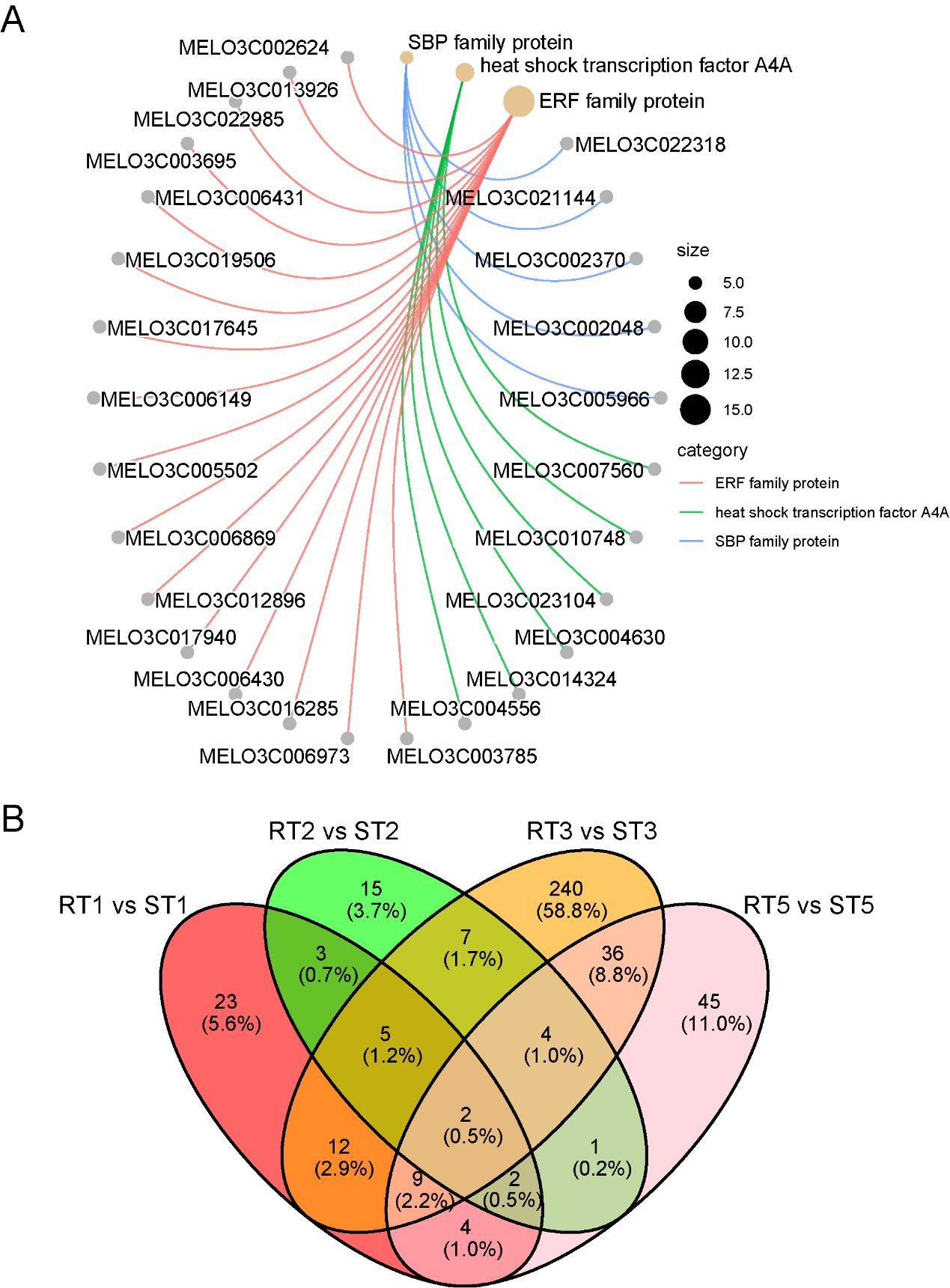


**Figure S7.** Analysis of transcription factor enrichment and Venn diagrams of differentially expressed transcription factors

(A) Transcription factor enrichment analysis of disease susceptible DEGs in ST1. Indicated is the enrichment analysis of differentially expressed transcription factors of TG5 on the first day after inoculation and 0 days before inoculation. (B) Venn diagrams of differentially expressed transcription factors between resistant material (TG1) and susceptible material (TG5) for the four periods. The abscissa RT1 vs ST1 represents the differentially expressed transcription factor between resistant material (TG1) and susceptible material (TG5) on the first day after inoculation with bacteria, and so on. The numbers in the Venn diagram represent the number of differentially expressed transcription factors, and the numbers in parentheses represent the percentage of the number of transcription factors in the total number.
